# Long-Term Follow-Up Results of Antiseizure Medication Withdrawal in Grade 2 and 3 Glioma Patients: A Prospective Observational Study

**DOI:** 10.1212/WNL.0000000000213590

**Published:** 2025-04-29

**Authors:** Pim B. van der Meer, Melissa Kerkhof, Linda Dirven, Marta Fiocco, Maaike J. Vos, Mathilde Kouwenhoven, Tjeerd J. Postma, Jacoline E.C. Bromberg, Martin J. Van Den Bent, Martin Taphoorn, Johan Koekkoek

**Affiliations:** 1 Department of Neurology, Leiden University Medical Center, the Netherlands;; 2 Department of Neurology, Haaglanden Medical Center, The Hague, the Netherlands;; 3 Department of Biomedical Data Sciences, Section Medical Statistics, Leiden University Medical Center, the Netherlands;; 4 Mathematical Institute, Leiden University, the Netherlands;; 5 Princess Maxima Center for Pediatric Oncology, Utrecht, the Netherlands;; 6 Department of Neurology, Amsterdam University Medical Center, the Netherlands; and; 7 Department of Neurology, Erasmus MC, Rotterdam, the Netherlands.

## Abstract

**Background and Objectives:**

The aim of this study was to evaluate the long-term results of seizure recurrence after antiseizure medication (ASM) withdrawal vs continuation in patients with diffuse glioma, grades 2 and 3.

**Methods:**

A prospective multicenter observational study was conducted, and patients were recruited from January 2014 until May 2016 from 3 neuro-oncology outpatient clinics in the Netherlands. The main inclusion criteria were as follows: history of ≥1 seizure, for which ASM was started; clinically and radiologically stable disease for ≥12 months; and seizure freedom for ≥12 months from the date of last antitumor treatment or seizure freedom for ≥24 months from the last seizure if seizures occurred after the last antitumor treatment. The primary outcome was time to recurrent seizure. A competing risk model was used to estimate cumulative incidences of recurrent seizure for ASM groups (i.e., ASM withdrawal vs ASM continuation) with death as the competing event. The proportional hazard assumption was violated for the ASM group; therefore, 2 Cox models were constructed for different time intervals (<48 months and ≥48 months since study inclusion).

**Results:**

A total of 71 patients were included (39 men [55%] and 58 older than 40 years [82%]); 46 patients with glioma (65%) were in the ASM withdrawal group and 25 (35%) in the ASM continuation group. The cumulative incidence of a recurrent seizure at 48 and 96 months was 48% (95% CI 33%–61%) and 66% (95% CI 48%–78%) for the ASM withdrawal group vs 28% (95% CI 12%–46%) and 52% (95% CI 31%–70%) for the ASM continuation group. The risk of a recurrent seizure differed in the 2 time intervals between the ASM continuation group (reference) and the ASM withdrawal group (cause-specific adjusted hazard ratio [aHR] 2.32 [95% CI 0.93–5.81], *p* = 0.071, during <48 months, and cause-specific aHR 0.73 [95% CI 0.21–2.49], *p* = 0.611, during ≥48 months since study inclusion).

**Discussion:**

Risk of recurrent seizure when withdrawing ASM was not statistically significantly higher in patients continuing ASM. However, a clinically relevant higher percentage of patients had a recurrent seizure in the ASM withdrawal group compared with the ASM continuation group. The lack of a statistical difference may be explained by the small sample size. Larger studies are needed to confirm these findings. Our results suggest that ASM withdrawal should be initiated cautiously and only when necessary.

**Classification of Evidence:**

This study provides Class III evidence that withdrawal of ASM does not significantly increase the risk of recurrent seizures in patients with glioma with stable disease and no seizures for >1 year. Confidence intervals do not exclude a clinically important increased risk of seizures.

## Introduction

One of the most common symptoms in patients with diffuse glioma is epileptic seizures, especially in patients with isocitrate dehydrogenase (IDH)–mutant gliomas who have a preoperative pooled incidence rate of seizures of 62%.^1^ Antitumor treatments, including surgical resection, radiotherapy, and chemotherapy, all have a beneficial effect on seizure control.^2^ Levetiracetam is considered first-choice antiseizure medication (ASM) for diffuse gliomas by most professionals in neuro-oncology,^3^ which has shown high efficacy and good tolerability and has no drug-drug interactions with chemotherapeutic agents temozolomide or procarbazine, lomustine, and vincristine.^4-6^ More variability in ASM prescription patterns arises if seizures remain uncontrolled on ASM monotherapy and ASM dual therapy is indicated. Commonly chosen add-on ASMs include lamotrigine, lacosamide, and valproic acid.^3^ Better seizure control with the ASM dual therapy combination levetiracetam with valproic acid was reported while tolerability was similar, compared with other ASM dual therapy combinations with either levetiracetam or valproic acid.^7^ In approximately 10% of diffuse glioma patients with epilepsy, ASM triple therapy is indicated because of insufficient seizure control on ASM dual therapy.^5^

As with all medications, ASM treatment is associated with a risk of (intolerable) adverse effects. In patients with glioma, ASM adverse effects are commonly of neurologic (e.g., headache), psychiatric (e.g., depression), or hematologic (e.g., low platelet count) origin. Adverse effects leading to ASM discontinuation by either the physician or the patient occur in 15%–20% of patients with glioma during 3 years of follow-up,^5,7^ but many patients suffer from adverse effects not leading to a change in ASM treatment.^8^ A risk-benefit evaluation is made by the physician weighing the potential benefits (improved seizure control) and disadvantages (adverse effects) of ASM treatment. For certain patients who have been seizure free for a considerable amount of time, the question arises whether the potential benefits still outweigh the harms of ASM treatment. No clear consensus exists about what the ideal period of seizure freedom is, but in non–brain tumor–related epilepsy (BTRE), a period of at least 1 or 2 years of seizure freedom is generally used for considering ASM treatment withdrawal.^9^ Few studies evaluating ASM withdrawal in patients with BTRE have been conducted. Two retrospective observational studies have been conducted, in which a recurrent seizure occurred in 19% (3/16) of adult brain tumor patients with epilepsy (the median follow-up after ASM withdrawal was 3.1 years) and in 27% (17/62) of child brain tumor patients (the median follow-up after ASM withdrawal was 2.3 years).^10,11^ Recently, a prospective observational study was conducted evaluating ASM withdrawal vs ASM continuation in grade 2 and 3 diffuse glioma patients with epilepsy. Patients with glioblastoma were not included, given their poor overall survival. A recurrent seizure occurred in 26% (12/46) in the ASM withdrawal group and in 8% (2/25) in the ASM continuation group (the median follow-up since study inclusion was 2.2 years for the ASM withdrawal group vs 1.7 years for the ASM continuation group).^12^ However, the long-term recurrent seizure rates after ASM withdrawal or continuation are currently unknown in patients with BTRE, although these are crucial in providing both patients and treating physicians with the information needed to make an informed decision whether to initiate ASM withdrawal or continue ASM treatment. This study reports the long-term results of seizure recurrence after ASM withdrawal or continuation in patients with grade 2 and 3 diffuse glioma within this study.^12^ We hypothesized, in line with our short-term results,^12^ that the risk of a recurrent seizure was higher for patients in the ASM withdrawal group compared with patients in the ASM continuation group.

## Methods

### Study Population and Procedures

The study methods have previously been published in the study protocol and original study report.^12,13^ In brief, a prospective multicenter observational study was conducted, and patients were recruited from January 2014 until May 2016 from 3 large referral neuro-oncology outpatient clinics in the Netherlands. Patients were consecutively screened for eligibility using the following inclusion criteria: (1) adults (18 years or older); (2) histologically confirmed World Health Organization (WHO) grade 2–3 diffuse glioma; (3) history of ≥1 epileptic seizure (excluding acutely provoked seizures), treated with ASM(s); (4) having undergone antitumor treatment (i.e., surgical resection, radiotherapy, and/or chemotherapy); (5) clinically and radiologically stable disease for ≥12 months; and (6) seizure freedom for ≥12 months since the date of the last antitumor treatment or seizure freedom for ≥24 months since their last seizure if seizures occurred after the last antitumor treatment. The treating neuro-oncologist (i.e., a neurologist with neuro-oncology as subspecialty) initially assessed whether it was safe for a patient to withdraw ASM treatment. If deemed safe, the decision to either withdraw or continue ASM was made jointly by the neuro-oncologist and the patient as part of a shared decision-making process. Both approaches (ASM withdrawal and continuation) are standard care for this patient population in our clinics. In case of any objection to withdraw ASMs, patients were included in the ASM continuation group and no changes were made to the ASM treatment regimen. In case patients were included in the ASM withdrawal group, dosage of each ASM was gradually decreased according to a fixed schedule (stepwise 50% dosage reduction every 2 weeks) and if ≥1 ASM, the last added ASM was withdrawn first. If a recurrent seizure occurred, ASM was restarted or dosage was increased according to the expertise of the treating neuro-oncologist.

Baseline information (i.e., from the starting date of study inclusion) was collected concerning sociodemographic characteristics, tumor (based on the WHO 2021 classification of tumors of the CNS) and antitumor treatment characteristics,^14^ radiologic progressive disease based on the Response Assessment in Neuro-Oncology criteria,^15^ and seizure characteristics. Standard follow-up in both groups took place after 3 months and subsequently every 6 months, until a patient died or was lost to follow-up. At each follow-up, data regarding ASM treatment, seizure frequency, and clinical and radiologic brain tumor progression were collected. Seizure recurrence was assessed based on patient history.

### Standard Protocol Approvals, Registrations, and Patient Consents

The institutional review board of each institution approved the protocol, and informed consent of all patients was obtained before inclusion in the study.

### Outcomes

The 2 primary outcomes for the original study, for which the results have already been published, were as follows: (1) the outcome of the shared decision making on ASM withdrawal or ASM continuation and (2) seizure freedom at 12 months and 24 months of follow-up. The primary outcome of this long-term follow-up study was time to recurrent seizure, from the starting date of study inclusion. Secondary outcomes were as follows: (1) time to restart ASM monotherapy because of seizures from the starting date of study inclusion, (2) time to start ASM dual therapy because of seizures from the starting date of study inclusion, and (3) time to start ASM triple therapy because of seizures from the starting date of study inclusion. ASM started for a different indication than seizures was not considered as restart ASM monotherapy, dual therapy, or triple therapy.

### Sample Size

The original study's primary aim was the shared decision-making process regarding ASM withdrawal or continuation, along with reasons behind these decisions. Therefore, no formal sample size calculation was made. A total of 100 patients with at least 30 patients in each group were planned to be recruited. Enrollment for this study was prematurely halted because of slow recruitment.

### Statistics

Baseline patient, tumor, antitumor treatment, and seizure characteristics were compared between the 2 groups. For categorical variables, the chi-square test was used, and for continuous variables (data were normally distributed), the independent *t* test was used. Differences in radiologic progression at the time of seizure recurrence, restart of ASM monotherapy, initiation of ASM dual therapy, and initiation of ASM triple therapy were compared descriptively between the 2 groups. Median progression-free overall survival (time since radiologic diagnosis) was estimated with the Kaplan-Meier methodology, and survival curves between the 2 groups were compared with the log-rank test. Median follow-up time (time since study inclusion) was calculated with the reverse Kaplan-Meier methodology. Time-to-event outcomes, including both primary and secondary outcomes, were analyzed using multivariable Cox proportional hazard models to assess the relationship between the ASM treatment group (ASM withdrawal vs continuation) and outcomes such as seizure recurrence. Potential confounders were included in the model. Death was considered a competing risk because it precludes the occurrence of events such as seizure recurrence.^16^ The following competing risk models were estimated: (1) recurrent seizure vs death; (2) restart of ASM monotherapy vs death; (3) start of ASM dual therapy vs death; (4) start of ASM triple therapy vs death.^17^ The proportional hazard assumption, which states that the hazard for each covariate does not change over time, was tested with the weighted residual test.^18^ This assumption was violated for the ASM treatment group, and thus, separate Cox models were estimated for 2 periods (<48 months and ≥48 months, since study inclusion). Based on simulation studies,^19^ ≥5 events per selected variable were required for the Cox proportional hazard model to ensure reliable results. We used the directed acyclic graph representation to identify potential confounders, based on previous knowledge from the literature and during discussion between authors. A confounder must be associated with both the determinant (i.e., ASM treatment withdrawal vs continuation) and the outcome (i.e., time to recurrent seizure) but it should not lie on the causal pathway between them. The following baseline characteristics based on preexisting knowledge were included as potential confounders in the Cox proportional hazard model for recurrent seizure: ASM treatment group (withdrawal vs continuation) tumor grade (2 vs 3), previous surgical resection (yes vs no), previous radiotherapy and/or chemotherapy (yes vs no), tumor involvement in the temporal lobe (yes vs no), seizure type (focal vs focal to bilateral tonic-clonic or combination of focal and focal to bilateral tonic-clonic), tumor progression before study inclusion (yes vs no), and ASM treatment before study inclusion (monotherapy vs polytherapy). Cause-specific adjusted hazard ratios (aHRs) were reported, which quantifies the risk of a specific event occurring at any given time, considering only those who have not yet experienced the event or a competing event (e.g., death). This measure is adjusted for potential confounders to account for differences in baseline characteristics between the 2 ASM treatment groups. The Gray test was used to assess the difference in the cumulative incidences between the 2 groups.^20^ The cumulative incidence represents the probability of a specific event occurring over time, accounting for competing risks (e.g., death). It reflects the proportion of patients who experience the event within a defined period. All statistical analyses were performed using SPSS version 25.0 and in the R software environment,^21,22^ and statistical significance was set at a *p* value of <0.05. Analyses regarding competing risk models were performed in R with the cmprsk package.^17^

### Data Availability

Data are available from the corresponding author on reasonable request.

## Results

### Patient Characteristics and Antitumor Treatment During Follow-Up

**Figure 1:**
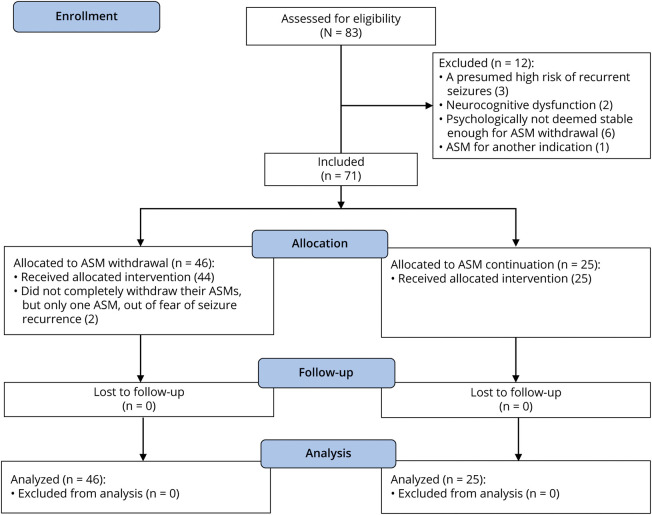
Flowchart of Patients in the Study ASM = antiseizure medication.

Figure 1 illustrates recruitment of patients in this study. Baseline characteristics of the 71 included patients with glioma are summarized in Table 1. Approximately 65% (46/71) were in the ASM withdrawal group and 35% (25/71) in the ASM continuation group. Main reasons to object ASM withdrawal by patients included risk of losing their driver's license if a recurrent seizure occurred and fear of recurrent seizures. Two patients in the ASM withdrawal group did not completely withdraw their ASMs, but only 1 ASM, out of fear of seizure recurrence, and remained subsequently on ASM dual therapy and ASM monotherapy. Patients were followed until December 2022.

**Table 1:** Demographic Characteristics of the Patients at Baseline

Characteristics	Group		
	ASM withdrawal	ASM continuation	*p* Value
Patients included, n (%)	46 (100)	25 (100)	
Age, n (%)			0.098
≤40 y	11 (24)	2 (8)	
>40 y	35 (76)	23 (92)	
Sex, n (%)			0.103
Male	22 (48)	17 (68)	
Female	24 (52)	8 (32)	
Tumor grade and pathology, n (%)^a^			0.662
Grade 2	27 (59)	16 (64)	
Diffuse astrocytoma, NOS	1 (2)	3 (12)	
Diffuse astrocytoma, IDH-mutant	13 (28)	2 (8)	
Oligodendroglioma, NOS	1 (2)	1 (4)	
Oligodendroglioma, IDH-mutant 1p/19q codeletion	11 (24)	10 (40)	
Oligoastrocytoma, NOS	1 (2)	0 (0)	
Grade 3	19 (41)	9 (36)	
Diffuse astrocytoma, NOS	4 (9)	5 (20)	
Diffuse astrocytoma, IDH-mutant	2 (4)	1 (4)	
Oligodendroglioma, IDH-mutant 1p/19q codeletion	12 (46)	3 (12)	
Oligoastrocytoma, NOS	1 (2)	0 (0)	
Surgical resection, n (%)			0.965
Yes	37 (80)	20 (80)	
No (including biopsy)	9 (20)	5 (20)	
Radiotherapy, n (%)			0.342
Yes	36 (78)	17 (68)	
No	10 (22)	8 (32)	
Systemic therapy, n (%)^b^			0.387
Yes	19 (41)	13 (52)	
Temozolomide	15 (33)	7 (28)	
PCV	6 (13)	6 (24)	
No	27 (59)	12 (48)	
Tumor progression before inclusion study, n (%)			0.555
Yes	10 (24)	7 (28)	
No	36 (76)	18 (72)	
Tumor involvement in the temporal lobe, n (%)			0.560
Yes	16 (35)	7 (28)	
No	30 (65)	18 (72)	
Seizure type, n (%)			0.157
Focal	10 (22)	3 (12)	
Focal to bilateral tonic-clonic^c^	30 (65)	14 (56)	
Unknown	6 (13)	8 (32)	
ASMs, n (%)^d^			0.239
ASM monotherapy	40 (87)	19 (76)	
Levetiracetam	25 (54)	10 (40)	
Valproic acid	9 (20)	5 (20)	
Carbamazepine	3 (7)	4 (16)	
Phenytoin	2 (4)	0 (0)	
Lamotrigine	1 (2)	0 (0)	
ASM polytherapy	6 (13)	6 (24)	0.239
Levetiracetam + valproic acid	2 (4)	1 (4)	
Levetiracetam + lacosamide	1 (2)	1 (4)	
Levetiracetam + clonazepam	0 (0)	1 (4)	
Levetiracetam + lamotrigine	0 (0)	1 (4)	
Levetiracetam + phenytoin	1 (2)	0 (0)	
Lamotrigine + valproic acid	0 (0)	1 (4)	
Lamotrigine + clobazam	1 (2)	0 (0)	
Levetiracetam + clobazam + lamotrigine	1 (2)	0 (0)	
Levetiracetam + clobazam + zonisamide	0 (0)	1 (4)	
ASM load, mean (SD)^e^	0.86 (0.57)	1.15 (0.74)	0.072

Abbreviations: ASM = antiseizure medication; IDH = isocitrate dehydrogenase; NOS = not otherwise specified; PCV = procarbazine, lomustine, and vincristine.

a Grade 2 vs grade 3.

b Does not add up to 100%, because 2 patients in the ASM withdrawal group received both temozolomide and PCV.

c Patients had either solely focal to bilateral tonic-clonic seizures or both focal and focal to bilateral tonic-clonic seizures.

d Monotherapy vs polytherapy.

e ASM load is defined as the sum of the ratio between the prescribed daily dosage and the defined daily dosage of ASMs.

Regarding baseline characteristics, no significant differences were found between the 2 groups. After study inclusion, in the ASM withdrawal group, 24% (11/46) of patients received surgical resection, 26% (12/46) radiotherapy, and 46% (21/46) chemotherapy (eTable 1). In the ASM continuation group, 12% (3/25) of patients received surgical resection, 24% (6/25) radiotherapy, and 48% (12/25) chemotherapy after study inclusion. During follow-up, occurrence of radiologic progressive disease did not differ between the ASM withdrawal group (54% [25/46]) and ASM continuation group (56% [14/25], *p* = 0.894). The median progression-free survival from radiologic diagnosis was 122.1 months (95% CI 96.5–147.8 months) for the ASM withdrawal group and 122.7 months (95% CI 102.4–142.9 months, *p* = 0.840) for the ASM continuation group. The median follow-up from study inclusion was 92.6 months (95% CI 90.8–94.5 months).

### Time to Recurrent Seizure

**Figure 2:**
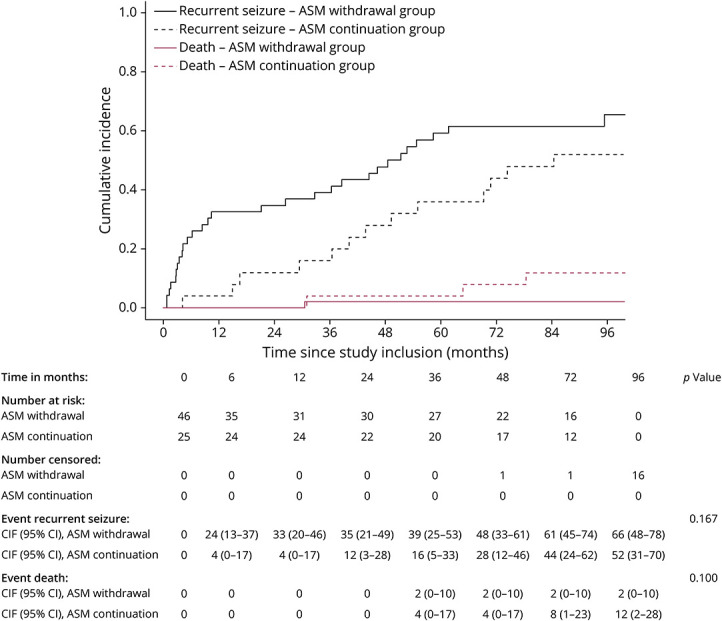
Time to Recurrent Seizure, From Moment of Study Inclusion ASM = antiseizure medication; CIF = cumulative incidence function.

During the total duration of follow-up, a recurrent seizure occurred in 63% (29/46) of patients in the ASM withdrawal group vs 52% (13/25) of patients in the ASM continuation group. The type of recurrent seizure in the ASM withdrawal group vs ASM continuation group was focal in 72% (21/29) vs 62% (8/13), focal to bilateral tonic-clonic in 24% (7/29) vs 23% (3/13), and unknown in 3% (1/29) vs 15% (2/13). The cumulative incidence of recurrent seizure at 48 months was 48% (95% CI 33%–61%) for the ASM withdrawal group vs 28% (95% CI 12%–46%, *p* = 0.167) for the ASM continuation group (Figure 2). Risk of recurrent seizure differed between the ASM withdrawal group and ASM continuation group (reference) with a cause-specific aHR of 2.32 (95% CI 0.93–5.81, *p* = 0.071) during <48 months since study inclusion and a cause-specific aHR of 0.73 (95% CI 0.21–2.49, *p* = 0.611) during ≥48 months since study inclusion (Table 2). The incidence of radiologic progressive disease at time of recurrent seizure was 24% (7/29) in the ASM withdrawal group and 46% (6/13) in the ASM continuation group.

**Table 2:** Unadjusted and Adjusted Hazard Ratios of Time to Recurrent Seizure, From Study Inclusion, With Death as a Competing Risk

Parameter	Recurrent seizure			
	uHR (95% CI)	*p* Value	aHR (95% CI)	*p* Value
ASM group,^a^ time since study inclusion <48 mo				
ASM continuation (ref)				
ASM withdrawal	2.14 (0.91–5.01)	0.80	2.32 (0.93–5.81)	0.071
ASM group, time since study inclusion ≥48 mo				
ASM continuation (ref)				
ASM withdrawal	0.84 (0.28–2.55)	0.755	0.73 (0.21–2.49)	0.611
Tumor grade				
Grade 2 (ref)				
Grade 3	1.07 (0.58–1.98)	0.832	1.39 (0.62–3.13)	0.431
Surgical resection before study inclusion				
No (ref)				
Yes	0.70 (0.34–1.42)	0.322	0.50 (0.20–1.24)	0.132
Radiotherapy and/or chemotherapy before study inclusion				
No (ref)				
Yes	0.81 (0.29–2.28)	0.690	0.58 (0.18–1.88)	0.360
Tumor involvement in the temporal lobe				
No (ref)				
Yes	1.22 (0.64–2.32)	0.548	0.85 (0.38–1.92)	0.693
Seizure type				
Focal (ref)				
Focal to bilateral tonic-clonic^b^	0.59 (0.29–1.19)	0.141	0.50 (023–1.09)	0.080
ASM treatment before study inclusion				
Monotherapy (ref)				
Polytherapy	1.83 (0.87–3.87)	0.111	2.05 (0.85–4.94)	0.109
Tumor progression before study inclusion				
No (ref)				
Yes	1.88 (0.95–3.71)	0.068	2.01 (0.88–4.57)	0.096

Abbreviations: aHR = adjusted hazard ratio; ASM = antiseizure medication; ref = reference; uHR = unadjusted hazard ratio.

a The proportional hazards assumption did not hold for the ASM group, and therefore, a time interaction was included.

b Patients had either solely focal to bilateral tonic-clonic seizures or both focal and focal to bilateral tonic-clonic seizures.

### Time to (Re)start ASM Treatment

**Figure 3:**
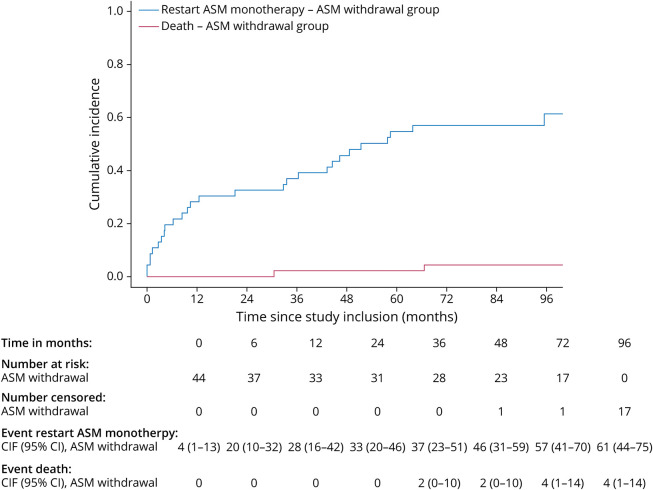
Time to Restart ASM Monotherapy, From Moment of Study Inclusion ASM = antiseizure medication; CIF = cumulative incidence function.

**Figure 4:**
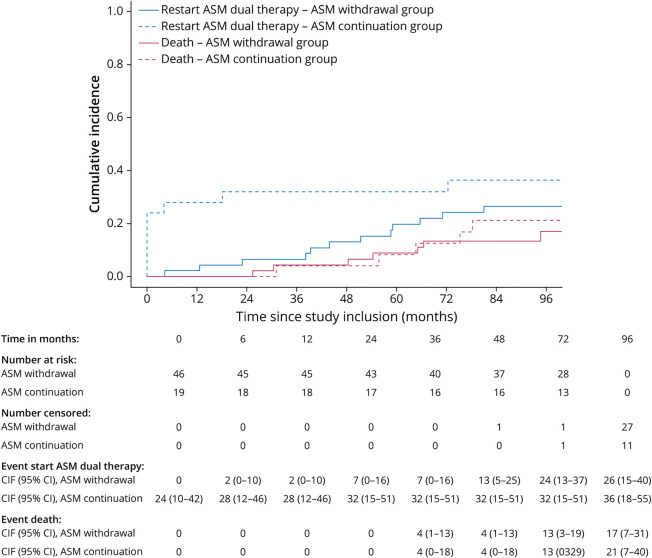
Time to Restart ASM Dual Therapy, From Moment of Study Inclusion ASM = antiseizure medication; CIF = cumulative incidence function.

**Figure 5:**
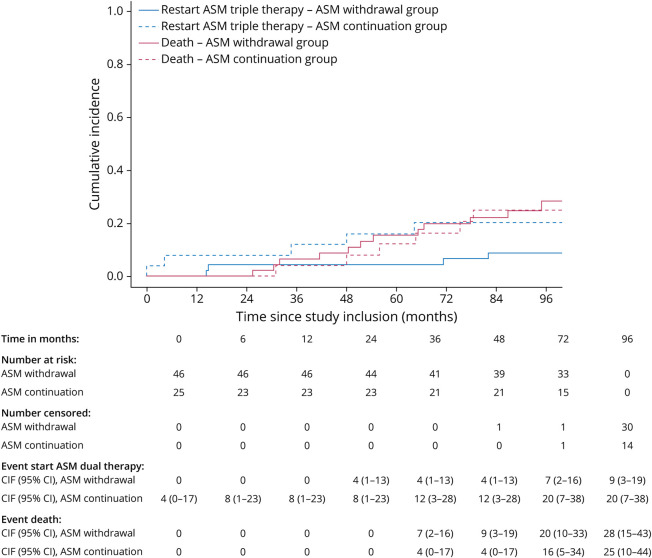
Time to Restart ASM Triple Therapy, From Moment of Study Inclusion ASM = antiseizure medication; CIF = cumulative incidence function.

Approximately 59% (27/46) restarted ASM monotherapy in the ASM withdrawal group while in the ASM continuation group, all patients continued their ASM at baseline. In the ASM withdrawal vs the ASM continuation group, 26% (12/46) vs 36% (9/25) started ASM dual therapy and 9% (4/46) vs 20% (5/25) started ASM triple therapy. The cumulative incidence of restart of ASM monotherapy after ASM withdrawal at 48 months was 46% (95% CI 31%–59%, Figure 3), for start of dual therapy in case of ASM withdrawal was 13% (95% CI 5%–25%, Figure 4) vs ASM continuation 32% (95% CI 15%–51%), and for start of triple therapy in case of ASM withdrawal was 4% (95% CI 1%–13%, Figure 5) vs ASM continuation 12% (95% CI 3%–28%). Presence of radiologic progressive disease at time to restart ASM monotherapy, dual therapy, and triple therapy can be found in eTable 2. No cause-specific HRs were estimated for restart of ASM monotherapy, start of ASM dual therapy, and start of ASM triple therapy, given the incomparability of the 2 groups regarding ASM treatment at baseline.

### Classification of Evidence

This study provides Class III evidence that withdrawal of ASM does not significantly increase the risk of recurrent seizures in patients with glioma with stable disease and no seizures for >1 year. Confidence intervals do not exclude a clinically important increased risk of seizures.

## Discussion

The aim of this prospective observational study was to evaluate the long-term follow-up results of ASM withdrawal in patients with diffuse glioma, WHO grades 2 and 3. In 96 months, recurrent seizure occurred in approximately two-thirds of patients withdrawing their ASM(s) and approximately half of the patients who were continuing their ASM. We did not find a statistically significant difference between the ASM withdrawal and ASM continuation group in time to recurrent seizure with death as a competing risk. However, a clinically relevant difference between the 2 groups was found during <48 months since study inclusion because patients withdrawing their ASM had a higher risk of recurrent seizure. Almost all patients restart ASM after the occurrence of a recurrent seizure, and during 96 months of follow-up in both groups, only a small minority of 9%–20% need ASM triple therapy because of uncontrolled seizures.

Results should be interpreted cautiously in our opinion because of the limitations of the study design and the clinically relevant difference in recurrent seizure between the 2 groups, especially during the first 48 months of follow-up. In this study, the percentage of patients having tumor progression at time of recurrent seizure was approximately twice as high in the ASM continuation group compared with the ASM withdrawal group. Nearly half of the patients in the ASM continuation group had tumor progression at time of recurrent seizure. In previous studies, tumor progression after initial surgical resection has been associated with risk of seizure recurrence in patients with glioma. Re-resection of the tumor can provide renewed seizure control in these patients.^23,24^ Based on these results, it seems that ASM does have a beneficial effect on seizure control in patients with glioma with well-controlled epilepsy, but a randomized study with sufficient statistical power is needed to confirm these preliminary results, preferentially with stratification by glioma molecular subtypes to better identify which patients may be suitable candidates for ASM withdrawal. Given the current evidence and the limitations of our study, ASM withdrawal should be considered cautiously and only in cases where there are compelling clinical reasons. The risk of a recurrent seizure and its consequences (e.g., mandatory driving suspension) has to be carefully weighed against the potential advantages of ASM withdrawal (e.g., reducing ASM-related adverse effects). At this stage, it may be premature to recommend ASM withdrawal as routine clinical care for glioma patients with epilepsy because our data suggest that continuing ASM could reduce the risk of recurrent seizures. To date, no placebo-controlled ASM efficacy studies have been conducted in patients with glioma.^4^ Owing to this lack of placebo-controlled studies, it is unclear to what extent ASMs are truly effective in patients with glioma.

Results from this long-term follow-up study are in line with the first results from the initial study,^12^ but an important difference is the change in risk of recurrent seizure between the 2 groups during the first 48 months and ≥48 months since study inclusion. During the first 48 months, the risk of a recurrent seizure is higher for patients in the ASM withdrawal group, while after the first 48 months, this risk becomes lower. This might be because the higher the risk of tumor progression, the longer the follow-up. Another explanation could be the reduced impact of glioma-related factors over time, such as tumor location or neurotransmitter imbalances. The 2 retrospective observational studies assessing risk of recurrent seizure after ASM withdrawal had a median follow-up of 2 and 3 years, which is much shorter than in our study, and found that 19%–27% of brain tumor patients with epilepsy had a recurrent seizure compared with 35% and 39% in our study at 2 and 3 years of follow-up, respectively.^10,11^ The lower risk of recurrent seizure in these studies might be attributable to differences in methodology and brain tumor entities included. ASM withdrawal may come with the possible risk that seizure control may be worse after restarting ASM treatment compared with before ASM withdrawal, which was seen in 19% (95% CI 15%–24%, mean of 14 studies) of patients in another study,^25^ and some might even develop treatment-resistant epilepsy.^26^ To what extent this has a cause-and-effect relationship is difficult to say. Approximately a quarter of patients in our study needed ASM dual therapy, and approximately 10% needed ASM triple therapy in the ASM withdrawal group. This means, even in this carefully selected group, some patients will eventually suffer considerably from their epilepsy again.

The chosen observational design, due to expected unwillingness of both physicians and patients to randomize, is a clear limitation of this study because it introduces confounding by indication (i.e., patients withdrawing ASM were inherently different from patients continuing ASM, because in the latter group, there were objections for ASM withdrawal either by the treating physician or patient). Despite no significant differences in baseline characteristics, this still might have affected differences in outcomes. In addition, residual confounding might be present, given that potential confounders such as specific tumor type were not controlled for, because molecular data such as IDH status were missing for a considerable proportion of patients. A proportion of these patients with missing IDH status might have been reclassified nowadays as a grade 4 glioma, which has a lower propensity to cause seizures. The extent of surgical resection is an important factor influencing seizure control. Unfortunately, we did not collect detailed data on residual tumor volume at baseline and only accounted for the potential confounder of surgical resection vs no resection in our analyses. The median residual tumor volume might have differed between the 2 groups and affected results. Although we included the most critical distinction in tumor location as a confounder (tumor involvement in the temporal lobe: yes vs no), a more detailed classification of tumor location(s) would have been preferable. However, the limited number of confounders that could be included in the analyses made this infeasible. Although n = 71 is a substantial number of included patients, given the rarity of the tumor type and the number of eligible patients, we acknowledge that the sample size was small. This resulted in reduced statistical power, wider CIs of our effect sizes, a higher risk of selection bias that may limit generalizability, and the increased risk of overfitting of the Cox models. Results cannot be easily generalized to higher grade gliomas, such as glioblastoma, given their worse overall survival, biological differences, different treatment approaches, and response to treatment. Owing to these study design issues, results should be interpreted cautiously. The inclusion criterion “seizure freedom for ≥12 months from the date of last antitumor treatment or seizure freedom for ≥24 months from the last seizure if seizures occurred after the last antitumor treatment” was derived from expert opinion. Although it is prudent to initiate ASM withdrawal only in patients after antitumor treatment, seizure recurrence risk seems relatively similar between patients with short-term, medium-term, and long-term seizure freedom after antitumor treatment.^27^ Given the limited prognosis of this patient population, ideally, the minimum period of seizure freedom is as low as possible (without increasing the risk of a recurrent seizure) if ASM withdrawal is initiated. The inclusion criterion regarding the period of seizure freedom might be too strict.

Our results suggest that most patients withdrawing their ASM have a recurrent seizure during 96 months of follow-up. Although we did not find a statistically significant difference between the ASM withdrawal group and ASM continuation group in time to recurrent seizure with death as a competing risk, we do believe that the higher risk of a recurrent seizure in the ASM withdrawal group is clinically relevant. Results should be interpreted cautiously because of the limitations of the study design, but in our opinion, ASM withdrawal should be initiated only if there are compelling reasons to do so because continuing ASM is preferred.

## Glossary

aHR: adjusted hazard ratio
ASM: antiseizure medication
BTRE: brain tumor–related epilepsy
IDH: isocitrate dehydrogenase
WHO: World Health Organization
